# Burnout Among Physicians of Specialties Dedicated to Liver Transplantation

**DOI:** 10.3389/ti.2024.13738

**Published:** 2024-11-14

**Authors:** Gloria Sanchez-Antolín, Gerardo Blanco-Fernández, Isabel Campos-Varela, Patricia Ruiz, José M. Álamo, Alejandra Otero, Sonia Pascual, Laura Lladó

**Affiliations:** ^1^ Department of Gastroenterology and Hepatology, Hospital Universitario Río Hortega, Valladolid, Spain; ^2^ Universidad de Extremadura, Facultad de Medicina y Ciencias de la Salud, Badajoz, Spain; ^3^ Department of Hepato-Pancreatic-Biliary Surgery and Liver Transplantation, Hospital Universitario de Badajoz, Badajoz, Spain; ^4^ Instituto Universitario de Investigación Biosanitaria de Extremadura (INUBE), Badajoz, Spain; ^5^ Department of Gastroenterology and Hepatology, Hospital Universitari Vall d’Hebron-Vall d’Hebron Research Institute (VHIR), Barcelona, Spain; ^6^ Hepatobiliary and Liver Transplantation Department, Hospital Universitario de Cruces, Bilbao, Spain; ^7^ Hepatobiliary and Liver transplantation Department, Hospital Universitario Virgen del Rocío, Sevilla, Spain; ^8^ Department of Gastroenterology and Hepatology, Hospital Universitario A Coruña, A Coruña, Spain; ^9^ Unidad Hepática, Hospital General Universitario Dr. Balmis, ISABIAL, CIBERehd, Alicante, Spain; ^10^ Hepatobiliary and Liver Transplantation Department, Hospital Universitario de Bellvitge, IDIBELL, University of Barcelona, Barcelona, Spain

**Keywords:** liver transplantation, burnout, physician, healthcare professionals, transplant teams

## Abstract

Burnout is increasingly relevant among healthcare professionals. The aim of this study is to describe the prevalence of burnout and other parameters of professional satisfaction among different specialists dedicated to Liver Transplantation (LT) in transplant teams. A working group from the Spanish Society of LT designed a survey with 39 questions evaluating the prevalence of parameters related to professional satisfaction, including burnout. It was distributed among 496 specialists dedicated to liver transplantation in Spanish transplant teams. Responders included surgeons (49%), hepatologists (27%), anesthesiologists (16%), intensivists (4%), and other specialties (4%). Among responders, 78% reported some degree of burnout. Moreover, 46% of responders did not see themselves working in transplantation in 5 years. The rates of burnout and dissatisfaction among anesthesiologists and surgeons were higher than other specialists. The highest levels of dissatisfaction were in economic remuneration and work–life balance. Being younger than 60 years old and non-head of department showed to be risk factors of burnout. In conclusion, the prevalence of burnout among LT physicians in Spain was notably high. Among the various specialties, anesthesiologists and surgeons exhibited the highest dissatisfaction rates. The results of this work may be of interest to healthcare management and planning.

## Graphical Abstract

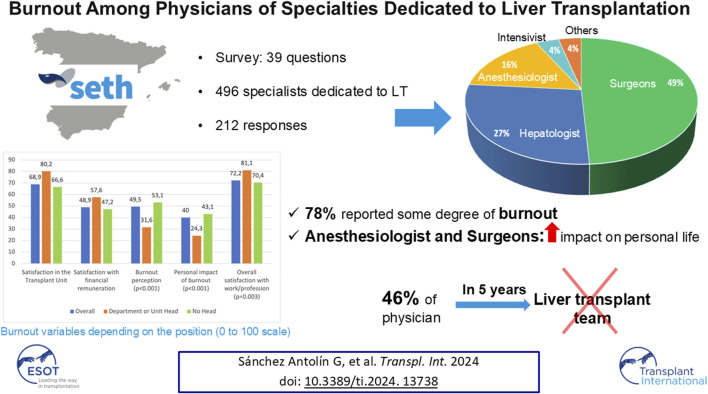

## Introduction

Burnout, first described in 1974 by Herbert Freudenberger [1], is a syndrome typically encountered in high-demand jobs [2]. The syndrome is increasingly relevant among healthcare professionals and is well-documented among surgeons, gastroenterologists, and hepatologists [3–6]. Burnout results in emotional exhaustion, loss of interest in work, depersonalization or lack of empathy toward patients and colleagues, and job dissatisfaction [7]. It is also associated with a higher risk of medical errors, sadness and, depression [8].

Liver transplantation (LT) is a complex treatment requiring high levels of professional competence from surgeons, hepatologists, anesthetists, intensivists, and other specialists. Some studies show that abdominal transplant surgeons are on call more nights per week than other surgical specialists and experience an alarmingly high prevalence of burnout and depression [9–11]. Hepatologists are also known to have high burnout rates due to work-time distribution, peer support, and affect [6]. Although burnout has been studied among anesthesiologists and intensivists, there is no research specifically related to LT [12, 13]. Overall, the existing literature on burnout has typically focused on its effects in specific specialties [14–17].

The aim of this study is to describe the prevalence of burnout and other parameters of professional satisfaction among different specialists dedicated to LT in Spanish transplant teams.

## Material and Methods

### Study Population

Participants were invited from medical specialists dedicated to LT, including surgery, hepatology, anesthesiology, intensive care, pediatrics, and pediatric surgery. The survey was sent to all members of Spanish Society of Liver Transplantation (SETH), and to achieve wider dissemination, the directors of transplant teams were contacted and asked to send the survey to specialists in their transplant units who were not members of SETH.

The survey was also sent to medical residents from the same specialty as the other staff doctors. They were residents in the specialties of general and digestive surgery, gastroenterology and hepatology, anaesthesiology and intensive care medicine.

### Survey Design

The Scientific committee of SETH designed a survey based on the questions of the Maslach model [18], adapted to the socio-labor structure of our environment, with 39 questions evaluating emotional exhaustion with a loss of interest in work, depersonalization or lack of empathy for patients and colleagues, and professional dissatisfaction. Questions were included about the impact of stress on professional life, personal life, and team support, as well as the approach to burnout and the attitude toward its therapeutic options. Personality refers to the subjective perception of personal character. The item “felt recognized” was referred to subjective feel of recognition of your own work by colleagues related and no related to LT.

### Survey Dissemination

Once designed and agreed upon by the working group, the survey was created using SurveyMonkey^1^. Personal or specific workplace data were not requested to ensure anonymity. An email was sent to eligible participants that included a cover letter explaining the purpose of the study, encouragement to participate, and a web link to the survey.

### Statistical Analysis

Descriptive statistics were estimated using frequencies (n) and percentages (%) for categorical data and means and standard deviations (SD) for continuous data. Differences between groups were analyzed using the non-parametric Mann–Whitney *U* test for quantitative variables, and differences between percentages or frequencies were assessed using Pearson’s chi-square test or Fisher’s exact probability test. A p-value <0.05 was considered significant. The IBM SPSS statistical software (version 22.0; IBM Corp., Armonk, NY) was used for calculations.

## Results

### Demographics

The survey was distributed to 496 physicians and had a 43% response rate (n = 212 responses). Of these, 78% (n = 165) were SETH members, with an even distribution between males and females (50.9% females), and a mean age of 45.17 ± 11.6 years (range 26–70 years). The mean age was significantly higher for males (48.6 ± 12.4 vs. 42.3 ± 10 years old (p = 0.00008). Of note, 30% of participants had been working in the same unit for >20 years and 44% had been in the same unit for <10 years. Nearly half of the respondents were surgeons (49%), followed by hepatologists (27.8%), anesthesiologists (16%), intensivists (3.8%), and others from various specialties (3.3%). Anesthesiologists (67.6%), intensivists (62.5%), and hepatologists (54.2%) were more often women, while only 42.3% were female surgeons (p = 0.101). Regarding the positions of respondents, 69.3% (n = 147) were attending physicians, 9.9% (n = 21) were residents, and 20.75% (n = 44) were department or unit heads. Only 20.5% of the heads were women. Overall 59%, 31%, and 6% of respondents worked in centers performing 21–50, 51–100, and >100 LTs annually.

### Satisfaction and Perception of Burnout

**FIGURE 1:**
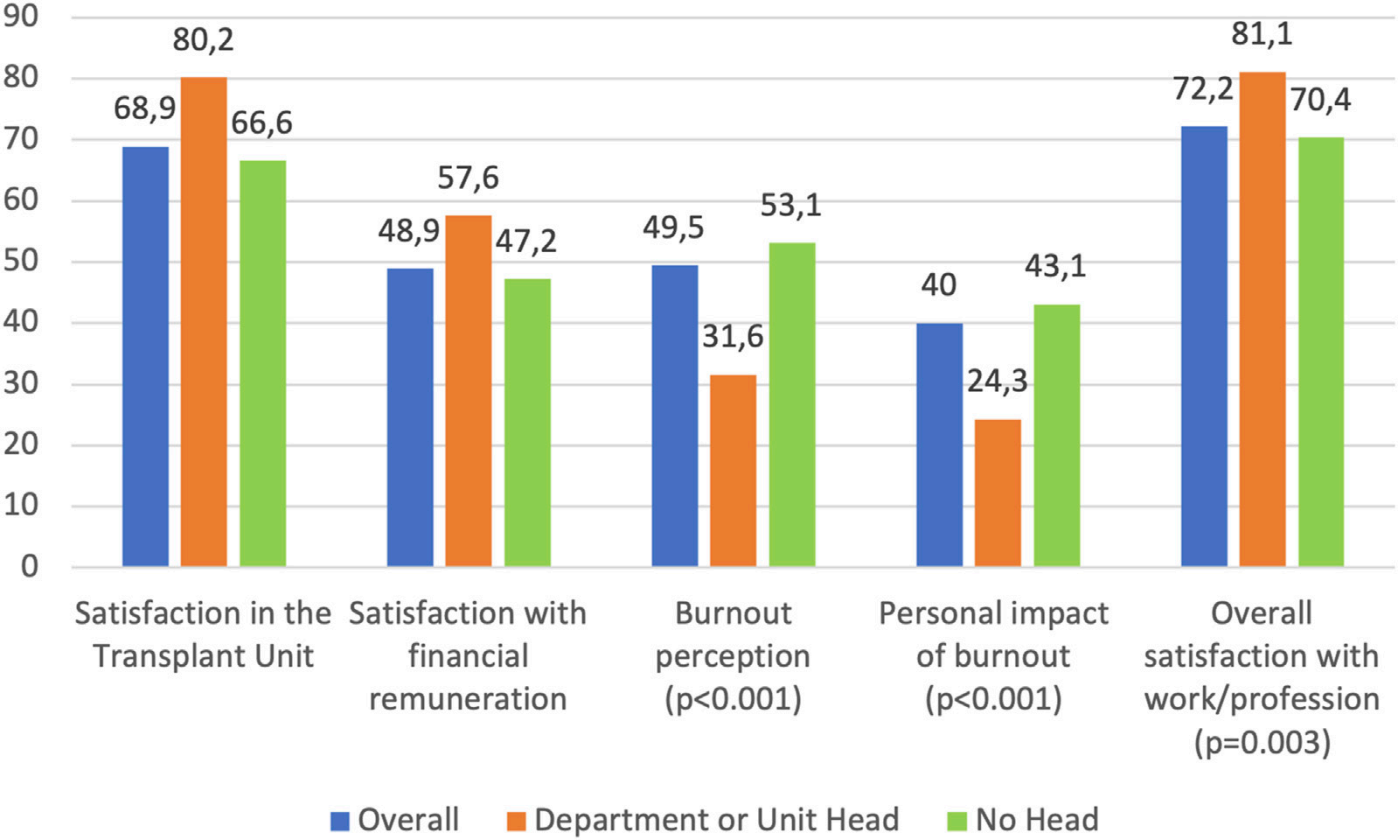
Burnout variables depending on the position (0–100 scale).

On a scale of 1–100, mean overall satisfaction with activities within the transplant unit was 68.86 (SD 24.3), while satisfaction with economic remuneration was the worst-rated aspect at 48.9 (SD 27.7) points. Department or unit heads were generally more satisfied and had a lower perceived burnout (Figure 1). Significant differences were observed in overall satisfaction, satisfaction with remuneration, perception of burnout, the consequences of burnout on life, and overall job satisfaction when analyzing data by specialty; notably, anesthesiologists and surgeons were most dissatisfied and had the highest reported burnout. These differences remained after excluding medical residents from the analysis (Table 1). Women also reported higher perceived burnout (55 ± 29.6 vs. 44 ± 32.9; p = 0.01) and impact on personal life (44.3 ± 26.9 vs. 35.6 ± 29.3; p = 0.02) (Table 2). Among respondents, 78% (n = 165) believed they suffered from some degree of burnout, all agreeing that it affected their work in some way. Regarding the impact on personal life, 27.4% (n = 58) reported that this was affected to a moderate-to-severe degree, with this especially common among women (33.6% vs 21%; p = 0.02) and surgeons (Table 3).

**TABLE 1:** Satisfaction and consequences of burnout according to specialty.

		Anesthesiology	Surgery	Hepatology	Intensive care	Others	Overall	p
Overall satisfaction (Media/Standar Desviation)	Residents and fellows included	60.4 (27.7)	65.5 (26.3)	75.4 (17)	85.4 (9.2)	85.4 (10.5)	68.9 (24.3)	0.001
	Residents and fellows excluded	60.2 (28.2)	66 (26.7)	76.3 (16.8)	85.4 (9.2)	85.4 (10.5)	69.6 (24.4)	0.001
Satisfaction with remuneration (Media/Standar Desviation)	Residents and fellows included	25.9 (23.1)	46.9 (29)	63.2 (20.1)	57.1 (16.4)	60.1 (11.4)	48.9 (27.7)	<0.001
	Residents and fellows excluded	26.1 (23.4)	49.8 (28.3)	63.6 (20.7)	57.1 (16.4)	60.1 (11.4)	50.4 (27.4)	<0.001
Perception of burnout (Media/Standar Desviation)	Residents and fellows included	59.4 (34.4)	52.4 (32.5)	45 (27.2)	31.5 (27.1)	22.7 (23.2)	49.5 (31.7)	0.0001
	Residents and fellows excluded	60.8 (34.1)	49.7 (32.5)	43.7 (27.5)	31.5 (27.1)	22.7 (23.2)	48.1 (31.7)	0.009
Consequences of burnout on life (Media/Standar Desviation)	Residents and fellows included	44.2 (30.3)	44.9 (29.7)	33.6 (23.2)	15.4 (17.7)	28.3 (26.5)	40 (28.4)	0.001
	Residents and fellows excluded	44.8 (30.6)	41.6 (29.1)	32.1 (23)	15.4 (17.7)	28.3 (26.5)	37.8 (27.9)	0.02
Overall satisfaction with your work (Media/Standar Desviation)	Residents and fellows included	66.7 (22.3)	69.6 (22)	76.8 (12.2)	81.1 (10.8)	89 (8.6)	72.2 (19.7)	0.0002
	Residents and fellows excluded	66.3 (22.5)	71 (21.8)	77.4 (12.1)	81.1 (10.8)	89 (8.6)	73.1	0.006

**TABLE 2:** Variables related to burnout according to sex.

Recognition with respect to others colleagues			
	Male n = 105	Female n = 107	p
Undervalued n (%)	4 (3.8%)	5 (4.7%)	0.07
Well recognized or highly recognized n (%)	77 (77.3%)	63 (58.9%)	
Neither recognized nor undervalued n (%)	24 (22.9%)	39 (36.4%)	
Satisfaction with work flexibility and family life balance			
	Male n = 105	Female n = 107	p
Dissatisfied or very dissatisfied n (%)	40 (38.1%)	52 (48.6%)	0.014
Satisfied or very satisfied … n (%)	42 (40%)	23 (21.5%)	
Neither satisfied nor dissatisfied n (%)	23 (21.9%)	32 (29.9%)	
Access to continued education and research and innovation tasks			
	Male n = 105	Female n = 107	p
Access to continued education never or almost never n (%)	21 (20%)	21 (19.6%)	0.5
Possibility of carrying out research or innovation tasks never or almost never n (%)	17 (6.2%)	34 (31.8%)	0.008
Work in the transplantation unit within 5 years (excluding residents and >60 years old)			
	Male n = 54	Female n = 78	
I will not be or I would like not to be n (%)	19 (26%)	8 (9.3%)	0.005

**TABLE 3:** Variables related to burnout according to specialty.

	Anesthesiology n = 34	Surgery n = 104	Hepatology n = 59	Intensive care n = 8	Others n = 7	Overall n = 212	p
Depression	4 (11.8%)	14 (13.5%)	5 (8.5%)	0 (0%)	1 (14.3%)	24 (11.3%)	0.7
Need for depression treatment	3 (8.8%)	6 (5.8%)	0 (0%)	0 (0%)	0 (0%)	9 (4.2%)	0.03
Burnout moderately or severely affects personal life	8 (23.5%)	36 (34.6%)	14 (23.7%)	0 (0%)	0 (0%)	58 (27.4%)	0.002
Influence of personality on the onset of burnout	1 (2.9%)	21 (20.2%)	15 (25.4%)	0 (0%)	1 (14.3%)	38 (17.9%)	0.02
You would like the institution to offer support for stress or burnout?	30 (88.2%)	79 (76%)	45 (76.3%)	7 (87.5%)	6 (85.6%)	167 (78.8%)	0.6
You would use support from the institution	13 (38.2%)	43 (41.3%)	29 (49.2%)	4 (50%)	3 (42.9%)	92 (43.4%)	0.8

Factors related to burnout that affected the personal life of respondents are shown in Table 4. Among the reported symptoms, tiredness was the most frequent (n = 74), followed by irritability (n = 47) and lack of motivation (n = 37). Depression was present in 24 (11.3%) participants, with 9 (4.2%) acknowledging a need for treatment. There were no differences between males and females in the rate of depression (12.4% vs. 10.3%; p = 0.6) or in acknowledging the need for treatment (2.9% vs 5.6%; p = 0.3). However, although there were no differences among specialties in the rate of depression, there were differences in acknowledging the need for treatment (Table 3).

**TABLE 4:** Factors associated with burnout that affect personal life.

	Burnout that affect personal life	p
Sex Male Female	72.4% 83.2%	0.058
Department or unit head Yes Not	51.4% 83.1%	0.000
Age <60 years Yes Not	81.7% 46.2%	0.000
Access to continued education Yes Never or almost never	73.5% 95.2%	0.002
Access to research Frequently Sometimes Never or almost never	68.1% 81.4% 90.2%	0.007
Specialty Anesthesiology Surgery Hepatology Intensive care Others	94.1% 78.8% 76.3% 37.5% 42.9%	0.001

**FIGURE 2:**
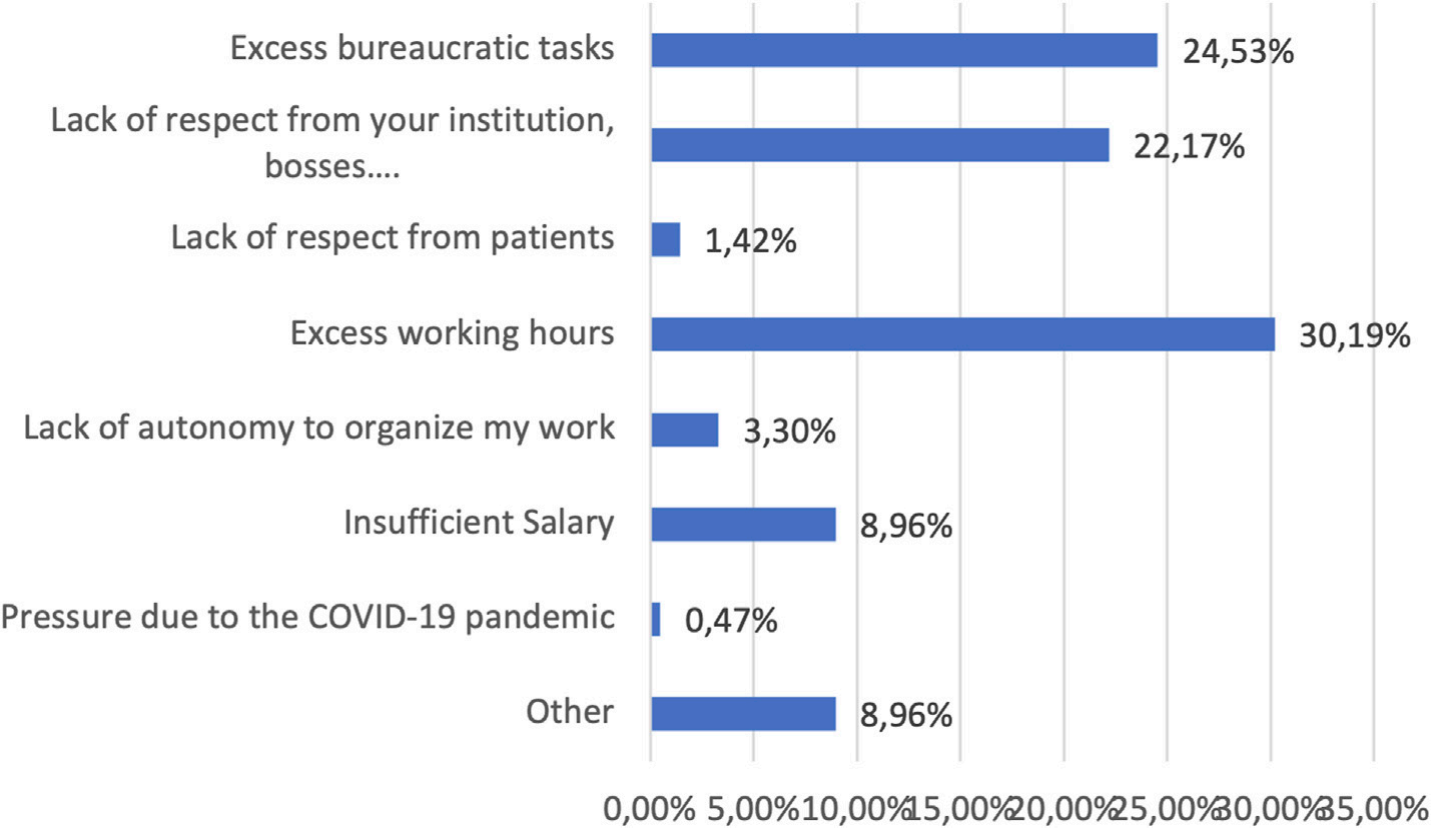
Frequency of factors associated with stress.

Regarding burnout management, 26.9% of participants (n = 57) sought support from family or friends, 34.9% (n = 74) turned to physical exercise, and only 4.2% (n = 9) reported needing the support of mental health services or pharmacological treatment. Although 78.8% (n = 167) reported that they would find it interesting if their institution offered support for stress or burnout, only 44% (n = 74) acknowledged they would use such a service, with no differences in response by gender or specialty (Table 3). Regarding the influence of personality on the onset of burnout, 10.9% (n = 23) were unsure if it affected them, 71.2% (n = 151) attributed their burnout to external factors, and 17.9% (n = 38) believed their personality played a role, with differences noted across specialties (Table 3). There were no differences between men and women (17.1% vs. 18.7%; p = 0.6). Factors recognized as affecting burnout were excessive working hours, excessive bureaucratic tasks, and lack of respect from the institution, bosses, and colleagues (Figure 2).

Univariate and multivariate analyses were conducted to assess variables associated with burnout. The results indicated that being younger than 60 years old (OR 2.89; 95% CI 1.09–7.61; p = 0.032), not being head of service or transplant unit (OR 4.14 95% CI 1.83–9.38; p = 0.001) and being an intensivist (OR 0.98 95% CI 0.021–0.448; p = 0.003) were significantly correlated with the risk of experiencing burnout (Table 5).

**TABLE 5:** Univariate and multivariate analysis of possible factors associated with burnout.

Variable	Univariate			Multivariate	
	Burnout	No Burnout	*p* < 0.05	OR (CI 95%)	OR (CI 95%)
Sex (female)	89 (83.2%)	18 (16.8%)	0.042		
SETH member	124 (75.6%)	40 (24.4%)	0.403		
Age <60 years	147 (81.7%)	33 (18.3%)	<0.001	2.89 (1.09–7.61)	0.032
Resident	21 (100%)	0	0.005		
No head	147 (83.1%)	30 (16.9%)	<0.001	4.14 (1.83–9.38)	0.001
Anesthesiologist	32 (94.1%)	2 (4.3%)	0.012		
Surgeon	82 (78.8%)	22 (21.2%)	0.744		
Intensivist	3 (37.5%)	5 (62.5%)	0.014	0.98 (0.021–0.448)	0.003
Hepatologist	44 (75.9%)	14 (24.1%)	0.712		
LT per year ≥ 50	63 (78.8%)	17 (21.3%)	0.867		

SETH, spanish society of liver transplantation; OR, odds ratio; LT, liver transplant.

Regarding to satisfaction with the work performed in the transplant unit (scale 0–100 points) we performed a multiple linear regression analysis and found that the independent factors related to this were: being a surgeon (correlation coefficient −0.257; p < 0.001), being an anesthesiologist (correlation coefficient −0.238; p = 0.001) and not being the head of the transplant unit (correlation coefficient −0.203; p = 0.002).

### Perception of Problems and Professional Recognition

**FIGURE 3:**
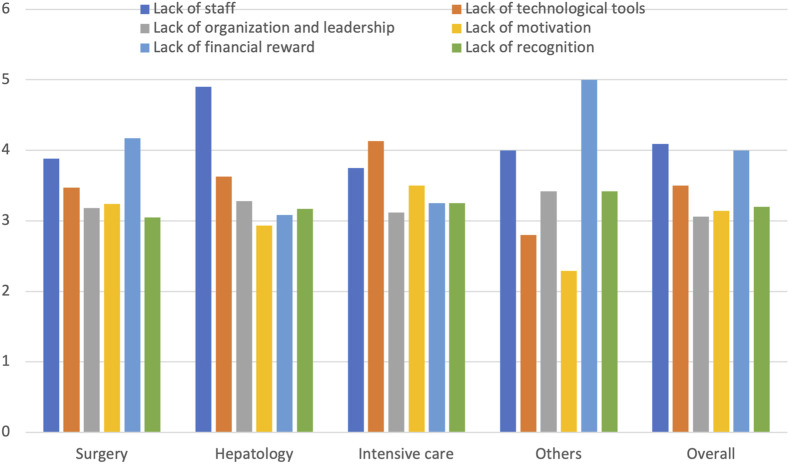
Importance of the most relevant problems depending on type of specialist.

When respondents were asked to rank issues within their transplant unit from least to most important on a scale of 1–5, three critical issues were identified: lack of personnel, lack of economic compensation, and lack of organization and leadership (Figure 3). Lack of the appropriate technological tools was the only factor perceived to be a more significant problem by men compared to women, with average scores of 2.77 ± 1.5 and 3.36 ± 1.5, respectively (p = 0.005). The other problems were rated similarly by both sexes. Differences were observed by specialty in the importance attributed to lack of economic reward, technological tools, and personnel (Table 6) (Figure 3). Additionally, compared with other physicians, department or unit heads considered the lack of technological tools more important (4.09 ± 1.36 vs. 2.86 ± 1.5; p < 0.001) and the lack of organization and leadership less important (2.68 ± 1.57 vs. 3.66 ± 1.86; p < 0.002). There was no significant differences whether they were women or men. Another noteworthy aspect is that heads respond that they can make their own decisions almost always or always more often than physicians who are not heads (80% vs. 45.8%; p < 0.001).

**TABLE 6:** Perception of the most important problems, professional recognition with respect to colleagues and access to continued education and research according to specialty.

	Anesthesiology n = 34	Surgery n = 104	Hepatology n = 59	Intensive care n = 8	Others n = 7	Overall n = 212	p
Perception of the most important problems^a^							
Lack of staff (media +/−SD)	3.44 ± 1.48	3.88 ± 1.78	4.9 ± 1.35	3.75 ± 1.67	4.0 ± 1.63	4.09 ± 1.68	<0.001
Lack of organization and leadership (media +/−SD)	3.35 ± 1.74	3.47 ± 1.97	3.63 ± 1.76	4.13 ± 1.81	2.86 ± 1.21	3.5 ± 1.84	0.6
Lack of technological tools (media +/−SD)	2.21 ± 1.22	3.18 ± 1.56	3.28 ± 1.51	3.12 ± 1.73	3.42 ± 1.72	3.06 ± 1.54	0.01
Lack of motivation (media +/−SD)	3.26 ± 1.44	3.24 ± 1.5	2.93 ± 1.67	3.5 ± 1.51	2.29 ± 1.6	3.14 ± 1.55	0.37
Lack of financial rewards (media +/−SD)	5.05 ± 1.58	4.17 ± 1.56	3.08 ± 1.65	3.25 ± 1.83	5.0 ± 1.15	4.0 ± 1.72	<0.001
Lack of recognition (media +/−SD)	3.67 ± 1.55	3.05 ± 1.59	3.17 ± 1.55	3.25 ± 2.12	3.42 ± 2.07	3.2 ± 1.61	0.4
Recognition with respect to others colleagues							
	Anesthesiology n = 34	Surgery n = 104	Hepatology n = 59	Intensive care n = 8	Others n = 7	Overall n = 212	p
Undervalued n (%)	1 (2.9%)	6 (5.8%)	2 (3.4%)	0 (0%)	0 (0%)	9 (4.2%)	0.001
Well recognized or highly recognized n (%)	12 (35.3%)	67 (64.4%)	49 (83.1%)	6 (75%)	6 (85.7%)	140 (66%)	
Neither recognized nor undervalued n (%)	21 (61.8%)	31 (29.8%)	8 (13.6%)	2 (25%)	1 (14.3%)	63 (29.7%	
Satisfaction with work flexibility and family life balance							
	Anesthesiology n = 34	Surgery n = 104	Hepatology n = 59	Intensive care n = 8	Others n = 7	Overall n = 212	p
Dissatisfied or very dissatisfied. n (%)	24 (70.6%)	55 (52.9%)	9 (15.3%)	2 (25%)	2 (28.6%)	92 (43.4%	<0.001
Satisfied or very satisfied n (%)	4 (11.8%)	29 (27.9%)	26 (44.1%)	3 (37.5%)	3 (42.9%)	65 (30.7%)	
Neither satisfied nor dissatisfied n (%)	6 (17.6%)	20 (19.2%)	24 (40.7%)	3 (37.5%)	2 (28.6%)	55 (25.9%)	
Access to continued education and research and innovation tasks							
	Anesthesiology n = 34	Surgery n = 104	Hepatology n = 59	Intensive care n = 8	Others n = 7	Overall n = 212	p
Access to continued education never or almost never n (%)	19 (55.9%)	18 (17.3%)	5 (8.5%)	0 (0%)	0 (0%)	42	p < 0.001
Possibility of carrying out research or innovation tasks never or almost never n (%)	17 (50%)	17 (16.3%)	15 (25.4%)	2 (25%)	0 (0%)	51 (24.1%)	0.02
Work in the transplantation unit within 5 years (excluding residents and >60 years old)							
	Anesthesiology n = 29	Surgery n = 70	Hepatology n = 48	Intensive care n = 7	Others n = 5	Overall n = 132	p
I will not be or I would like not to be	10 (34.5%)	13 (18.6%)	4 (8.3%)	0 (0%)	0 (0%)	27 (17.0%)	0.001

^a^ 1 (least important) to 6 (most important).

Assessment of time constraints revealed high levels of clinical pressure among 25.5% of participants (n = 54), who felt they almost never had enough time to perform their tasks well; another 43.9% (n = 93) felt this way sometimes. Of the 212 respondents, 33% (n = 70) felt their opinions were not considered within the team, though they were allowed to express them, and 30.7% (n = 65) believed their achievements were never or almost never recognized. Despite 4.25% (n = 9) feeling undervalued compared to other physicians not involved in LT, most (66%) felt recognized or highly recognized, although this sentiment varied by specialty (Table 6). None of the department or unit heads felt undervalued, as compared with 5.1% of other professionals. Likewise, 94.3% of department or unit heads felt recognized or very recognized compared with 60.5% of other professionals (p = 0.02). No statistically significant differences were found by gender. Colleague support was present always or almost always for 65% of respondents.

### Continued Education, Performance, and Professional Future

Regarding decision-making in professional performance, 51.4% (n = 109) had the ability to make decisions and only 45.8% (n = 98) believed their work was well organized almost always or always. Concerning opportunities for professional development, 29.7% (n = 63) were dissatisfied or very dissatisfied. Up to 43.4% (n = 92) were dissatisfied or very dissatisfied with work flexibility and family life balance. This parameter showed the highest dissatisfaction levels among anesthesiologists (70.6%) and the lowest levels among hepatologists (15.3%; p < 0.001) (Table 6). By gender, women reported higher rates of being dissatisfied or very dissatisfied with work flexibility and family life balance (48.6% vs. 38.1%; p = 0.014). We carried out a multivariate analysis to study which variables influence dissatisfaction with work-life balance, we found that gender was not a statistically significant variable, however, the following variables had a greater influence on dissatisfaction: Not being head of service or head of a department (OR 4.119 CI 95% 1.624–10.45; p = 0.003), being anesthesiologist (OR 10.663 CI 95% 4.073–27.917; p < 0.001) or surgeon (OR 5.948 CI 95% 2.862–12.362; p < 0.001).

Continued education was seen as always or almost always accessible for 42% of respondents (n = 89), while 19.8% (n = 42) admitted they never or almost never had access. No gender differences were detected (Table 2), but variations were observed by specialty, with anesthesiologists reporting the least access (Table 6). A similar pattern emerged for participation in research and innovation, with 24.1% (n = 51) stating they never or almost never had the opportunity to participate. Differences were observed among specialties, with anesthesiologists again reporting the least access (Table 6). Additionally, twice as many women as men reported that access to research and innovation was never or almost never possible (16.2% vs. 31.8%; p = 0.008) (Table 2).

Overall, 147 respondents (69.3%) reported that going to work was satisfying or very satisfying, and 104 (49%) considered their working conditions good or very good. Despite the challenges faces, 91% of transplant physicians felt committed or very committed to their work.

Regarding their futures within transplant teams, 54.3% of physicians (n = 115) saw themselves as part of the team in 5 years, while 21.2% (n = 45) would either not be or prefer not to be in the team. Importantly, 24.5% (n = 52) expressed a desire to stay, but they were unsure if they could withstand the pressure. To refine the analysis of how many physicians did not wish to remain in the transplant unit within 5 years, we excluded residents and those over 65 years of age. Among the remaining 132 respondents, 27 (17%) indicated they would not want to be part of the team. This percentage was higher among men than women (26% vs. 9.3%; p = 0.005); among the different specialties, it was highest among anesthesiologists (34.5%; p = 0.001) (Table 6). Among the surgery residents, it was notable that 27.8% thought they would not work or would not like to work in a LT unit.

## Discussion

In this survey of principal medical specialties involved in LT, we found that burnout was present in 78%, a rate that is higher than previously reported [3–6]. The high rate of burnout, especially among anesthesiologists and surgeons, was associated with lack of support and recognition from the team, superiors, and the institution. The greatest dissatisfaction centered on economic incentives, especially for anesthesiologists and surgeons.

We designed an adapted burnout survey for transplant doctors, rather than using a validated one like the MBI, to better capture the specific challenges of transplant work, such as the emotional and organizational pressures unique to this field. Additionally, the survey is tailored to the Spanish healthcare context and offers greater flexibility to address specific factors like workload in transplant units and lack of resources. Importantly, the survey maintains the structure of a validated tool by assessing key dimensions such as emotional exhaustion, depersonalization (or cynicism), and reduced personal accomplishment, ensuring that it covers the core aspects of burnout. The goal is to obtain practical and immediate results that help implement targeted interventions to improve the wellbeing of the team.

A 43% response rate in a burnout survey among transplant doctors is acceptable for this type of population. While it isn't high, it’s common for surveys in busy professional groups like doctors, where response rates typically range from 30% to 60%. Although a higher rate would be ideal, this level of participation can still yield valuable insights.

A 2015 national survey among transplant surgeons in the United States showed high levels of emotional exhaustion (40.1%), depersonalization, and low personal satisfaction. Lack of autonomy in decision-making, lack of support from superiors, and high patient demands were associated with higher levels of burnout [19]. A study of burnout among abdominal transplant surgeons in Europe also found that nearly a third exhibited emotional exhaustion, but that levels of depersonalization were low, suggesting that commitment to their work remained despite feeling exhausted [20]. Our data support the importance of physician commitment to their work, with 91% of respondents feeling committed or very committed.

Among intensivists, severe burnout has been described at rates of up to 50% [21]. Although dissatisfaction in our series was lower than that of anesthesiologists, surgeons, and hepatologists, the low number of participants mean that our results should be interpreted with caution. Studies among anesthesiologists show one of the highest prevalences of burnout, with higher rates of suicide and addiction than in the general population. Autonomy, control of the work environment, professional relationships, leadership, and organizational justice are considered the most important factors in job satisfaction [22].

A factor associated with dissatisfaction in our study was the difficulty maintaining a work–life balance, especially for anesthesiologists, consistent with the results of other studies [22]. It is also noteworthy that dissatisfaction with work–life balance was higher among women involved in LT, although this variable was not statistically significant in the multivariate analysis. A systematic review exploring the influence of gender on physician burnout found that both men and women experience high rates of burnout, but that it is more likely to develop in females, especially emotional exhaustion [23].

The rate of perceived burnout did not change when excluding medical residents from the analysis, suggesting they are affected similarly to other physicians. A study conducted among surgical transplant residents in the United States found that up to 17% exhibited symptoms of burnout, and that those working >100 h per week were more likely to experience severe stress, contemplate leaving their residency, or commit a medical error [2]. High levels of burnout and suicide have also been described among medical trainees in intensive care and anesthesiology [24]. West et al. has reported that physician burnout leads to dysfunction in the healthcare system by losing organizational talent, reducing patient care quality, and ultimately causing severe mental health damage to professionals [25].

It was notable that almost half of the respondents felt that they could not make decisions within the team, and that only 46% felt that their work was well organized always or almost always. The value given to organizational factors and decision-making might explain the lower perceived rate of burnout among service or unit heads, who can make these decisions. In public hospitals in Spain, department heads typically work around 37.5 h per week, not including on-call shifts. Although there is no fixed national regulation, it is common for 20%–30% of this time to be reserved for management duties, such as resource planning and team coordination. This protected time can vary depending on the autonomous community or the hospital, and may also depend on the clinical workload of the department. The regulation of this time is often governed by specific labor agreements in each region.

Regarding burnout management, it was striking that only 4.2% of respondents had sought professional help, and that, despite recognizing institutional support as interesting, only 35% would use it if implemented by their institution. We have no information on why professionals would not use support to treat or prevent burnout even if their institution provided it. This is likely due to fear of being labelled or fear of losing anonymity. This could be a relevant aspect for future research. The literature also highlights low adherence by physicians to support programs, which is considered to reflect their tendency to care for others but not themselves [26]. Moreover, there is little evidence of their benefit, and given the complexity of implementing preventive measures due to the heterogeneity of workers and the causes of workplace stress, results cannot be extrapolated [27, 28].

Another finding was that up to a quarter of participants acknowledged not having access to research, with concern that this issue presents twice as much in women compared to men. A recent study analyzing authorship of published papers between 2012 and 2021 in the United States observed that, despite an increase in women as first or last authors, there is still a significant gender gap. However, a female last author is associated with the presence of a female first author, highlighting the importance of mentoring young women entering transplantation [29]. Regulated continuous education and mentorship are considered essential to ensure the generational replacement of physicians [30].

Our results indicate that approximately 21% of respondents recognize that they will not be, or would not like to be, working in LT in 5 years. This is especially worrying in the case of residents, where the percentage rises to 27.8%. A study among surgery residents in Spain showed that most surgery residents did not want to dedicate themselves to transplantation because they considered the specialism too demanding [31]. This lack of motivation to dedicate themselves to transplantation has been highlighted by other authors [32–34].

As limitations of our study, we highlight that it relied on self-perception and lacked standardization, with bias toward a higher response rate among professionals more sensitized to burnout. Furthermore, the characteristics of the Spanish healthcare system are probably associated with greater dissatisfaction, due to the low salaries of professionals, which may complicate comparisons with series from other countries.

The survey did not include descriptive variables. The main reasons of dissatisfaction were remuneration, and work-life balance specially in women; thus, we may conclude that these are the reasons why physicians would not want to be in LT in the future.

Salary comparisons across specialties cannot be performed because we have no specific no data about remuneration for LT among the different specialist in Spain. This fact is not regulated in Spain, and each hospital, and each department has his own rules. Physician’s salaries are among the lowest in Europe, but not only in the transplant setting [35]. Regulation of salary and comparison across specialties in Spain with international teams may help to mitigate dissatisfaction. Parallelly to regulation of salaries, several regulatory, phycological and institutional solutions should be implemented at individual and organization-level [25]. Effective solutions should align with the drivers described at our study. Due to excessive workload, and low ratio of physicians, in most Spanish LT departments, research is not clearly scheduled, and there is no protected time to do it. This is clearly a field to be improved. Another limitation of our study is that physicians of the same specialty who do not work in transplantation teams have not been surveyed in order to make objective comparisons. Comparisons were based on the self-perception of the respondents. Nevertheless, this study has several strengths: the response rate was high, it covered the whole of Spain (a country with consolidated experience in LT), and included all major specialties involved in LT together, and not in isolation. The results of this work may be of interest to healthcare management and planning.

The high degree of burnout among LT physicians is the main conclusion of our study, and we consider it to be a warning to all healthcare stakeholders, especially the responsible of healthcare organizations. We should implement all needed interventions to improve the degree of burnout and mitigate dissatisfaction. It is imperative to avoid the decreasing number of professionals dedicated to LT, and evermore to avoid an increase of adverse events and effects on patients care that are related to burnout [25].

Previous studies have reported possible solutions to improve the degree of burnout and their outcomes. Efforts may be focused on salary, job security and flexibility, protected workload and professional development [25, 36]. The results of our survey suggest that healthcare system leaders and hospital administrators should implement strategies, not only economic ones, to minimize burnout among transplant professionals. These strategies should focus on increasing professional recognition, improving work-life balance, facilitating career progression, reducing excessive workloads, and providing emotional and psychological support.

In conclusion physicians dedicated to LT in Spain show high levels of commitment to their work. However, burnout rates were high (78%), being among anesthesiologists and surgeons higher than those of other specialists involved in LT. The highest levels of dissatisfaction were experienced for the perceived economic remuneration and the impact on balance with family life, with the latter especially common among women.

## Data Availability

The raw data supporting the conclusions of this article will be made available by the authors, without undue reservation.

## Footnotes

1
www.surveymonkey.com

## Abbreviations

LT, Liver transplantation; SETH, Spanish Society of Liver Transplantation.
