# Supplementary material for: Burnout Among Physicians of Specialties Dedicated to Liver Transplantation
Source: Transpl Int. 2024 Nov 14;37:13738. doi: 10.3389/ti.2024.13738 (PMC11602281; doi:10.3389/ti.2024.13738)
Supplement: Supplementary file 1 [file DataSheet1.pdf]

SurveyMonkey

Burnout Survey –
Spanish Society of Liver Transplantation (SETH)

1. Are you a member of SETH?: ['YES', 'NO']

2. I am: ['Woman', 'Man']

3. My age is: ['age in number']

4. I am: ['Resident', 'Attending Physician', 'Section Chief', 'Service Chief', 'Other']

5. My specialty is: ['Surgery', 'Hepatology', 'Anesthesia', 'Intensive Medicine', 'Other']

6. How many years have you worked in the Unit?: ['<5', '5-10', '11-15', '16-20', '21-25', '26-30', '>30']

7. How many transplants are performed on average in your Unit?: ['<20', '21-50', '51-100', '>100']

8. What is your overall satisfaction with your activity in the Transplant Unit, where 0 is very poor and 100 is excellent?: ['Ranking from 0 to 100']

9. In your opinion, what are the most important problems of the Unit (Rank from most to least important)?: ['Lack of staff', 'Lack of technological tools', 'Lack of organization and leadership', 'Lack of motivation', 'Insufficient economic reward', 'Lack of recognition within the hospital']

10. Going to work currently feels: ['Very enjoyable', 'Enjoyable', 'Indifferent', 'Unpleasant', 'Very unpleasant']

11. My working conditions are: ['Very good', 'Good', 'Acceptable', 'Bad', 'Very bad']

12. My ability to contribute to improving the Unit's organization is: ['Very high', 'High', 'Average', 'Low', 'Very low']

13. I feel that my achievements are recognized in the Unit: ['Always', 'Frequently', 'Sometimes', 'Rarely', 'Never']

14. How do you feel about your colleagues who are not involved in the transplant unit?: ['Very well', 'Well', 'Indifferent', 'Bad', 'Very bad']

15. I am satisfied with the professional development opportunities offered to me: ['Very satisfied', 'Satisfied', 'Indifferent', 'Dissatisfied', 'Very dissatisfied']

16. I am satisfied with the work-life flexibility in my Unit: ['Very satisfied', 'Satisfied', 'Indifferent', 'Dissatisfied', 'Very dissatisfied']

17. I feel committed to my job: ['Completely', 'A lot', 'Somewhat', 'A little', 'Not at all']

18. I have enough time to do my job well: ['Always', 'Frequently', 'Sometimes', 'Rarely', 'Never']

19. I can make decisions related to my work: ['Always', 'Frequently', 'Sometimes', 'Rarely', 'Never']

20. Is my work well organized?: ['Very well organized', 'Well organized', 'Acceptably organized', 'Poorly organized', 'Very poorly organized']

21. The leadership of my unit: ['Is very good', 'Is good', 'Is acceptable', 'Is bad', 'Is very bad']

22. Communication between the team leader and the rest of the team is: ['Very good', 'Good', 'Acceptable', 'Bad', 'Very bad']

23. I have access to continuing education: ['Always', 'Frequently', 'Sometimes', 'Rarely', 'Never']

24. In my unit, I can participate in research and innovation tasks: ['Always', 'Frequently', 'Sometimes', 'Rarely', 'Never']

25. I am satisfied with my remuneration (Where 0 is not at all satisfied and 100 is fully satisfied): ['Ranking from 0 to 100']

26. My colleagues take the initiative to help each other when necessary: ['Always', 'Frequently', 'Sometimes', 'Rarely', 'Never']

27. The most important incentives for me would be (Rank from 1, the most important, to 5, the least important): ['Economic', 'Recognition of my work by my institution', 'Recognition of my work by my patients', 'Recognition of my work by my boss', 'Recognition of my work by my colleagues']

28. Do you consider yourself "burned out," meaning your job causes anxiety, nervousness, or sadness?: ['Yes', 'No']

29. What do you think is the biggest factor contributing to stress in your job?: ['Excess of bureaucratic tasks', 'Lack of respect from your institution', 'Lack of respect from patients', 'Excessive working hours', 'Lack of autonomy to organize my work', 'Insufficient salary', 'Pressure from the COVID-19 pandemic', 'Other (specify)']

30. Do you think other team members experience the same "burnout" as you?: ['They have more', 'They have less', 'They have the same']

31. Do you think your personality contributes to experiencing more or less burnout?: ['Yes, more', 'Yes, less', 'I am not sure']

32. Do you think you are depressed?: ['No, not at all', 'No, but I am stressed', 'Yes, but it does not affect me much', 'Yes, and I need emotional support']

33. How significant is your burnout?: ['It affects my professional life but does not affect my personal life', 'It affects my work and slightly affects my personal life', 'It affects my work and moderately affects my personal life', 'It affects my work and seriously impacts all aspects of my personal life', 'I do not have burnout']

34. How does burnout affect you?: ['It does not affect me', 'I am frequently tired', 'I am slightly unmotivated', 'I am irritable and unmotivated', 'I am depressed and lack the desire to work']

35. On a scale of 0 (none) to 100 (a lot), how much does burnout affect your personal life, including relationships with your partner, family, and friends?: ['Ranking from 0 to 100']

36. What do you do to reduce stress?: ['Exercise', 'Family and friends relationship', 'Seeking help from mental health professionals', 'Nothing', 'I need medication', 'Two or more of the above answers']

37. Would you like your hospital to offer support for stress and burnout?: ['Absolutely, and I would use it', 'Absolutely, but I would not use it', 'I do not think it is necessary', 'I do not know']

38. Do you see yourself in the transplant team in five years?: ['Definitely yes', 'Definitely no', 'I would like to stay but I am unsure if I can handle the pressure', 'I would prefer to leave but do not think I can due to limited job opportunities', 'My personal situation does not allow me to change units']

39. Overall, my satisfaction with my job is (0 none, 100 maximum): ['Ranking from 0 to 100']

40. Would you like to add any comments?: ['Open response']
